# Neuroprotective Effects of Thiamine and Precursors with Higher Bioavailability: Focus on Benfotiamine and Dibenzoylthiamine

**DOI:** 10.3390/ijms22115418

**Published:** 2021-05-21

**Authors:** Margaux Sambon, Pierre Wins, Lucien Bettendorff

**Affiliations:** Laboratory of Neurophysiology, GIGA-Neurosciences University of Liège, 4000 Liège, Belgium; margaux.sambon@gmail.com (M.S.); winspierre9@gmail.com (P.W.)

**Keywords:** thiamine diphosphate, neurodegeneration, neuroprotection, Alzheimer’s disease, oxidative stress, diabetes, inflammation, glutathione, glycogen synthase kinase-3, transketolase

## Abstract

Thiamine (vitamin B1) is essential for brain function because of the coenzyme role of thiamine diphosphate (ThDP) in glucose and energy metabolism. In order to compensate thiamine deficiency, several thiamine precursors with higher bioavailability were developed since the 1950s. Among these, the thioester benfotiamine (BFT) has been extensively studied and has beneficial effects both in rodent models of neurodegeneration and in human clinical studies. BFT has antioxidant and anti-inflammatory properties that seem to be mediated by a mechanism independent of the coenzyme function of ThDP. BFT has no adverse effects and improves cognitive outcome in patients with mild Alzheimer’s disease (AD). Recent in vitro studies show that another thiamine thioester, dibenzoylthiamine (DBT) is even more efficient that BFT, especially with respect to its anti-inflammatory potency. Thiamine thioesters have pleiotropic properties linked to an increase in circulating thiamine concentrations and possibly in hitherto unidentified metabolites in particular open thiazole ring derivatives. The identification of the active neuroprotective derivatives and the clarification of their mechanism of action open extremely promising perspectives in the field of neurodegenerative, neurodevelopmental and psychiatric conditions.

## 1. Introduction

Thiamine (vitamin B1), the first vitamin to be isolated [1], is an essential micronutrient for all animal species. Thiamine deficiency (TD) is a life-threatening condition that, at least in vertebrates, causes various disorders and lesions in nerves and brain. Early investigations, starting at the end of the 19th century, led to the discovery that chronic dietary TD caused the appearance of a polyneuritic syndrome called beriberi [2].

**Figure 1:**
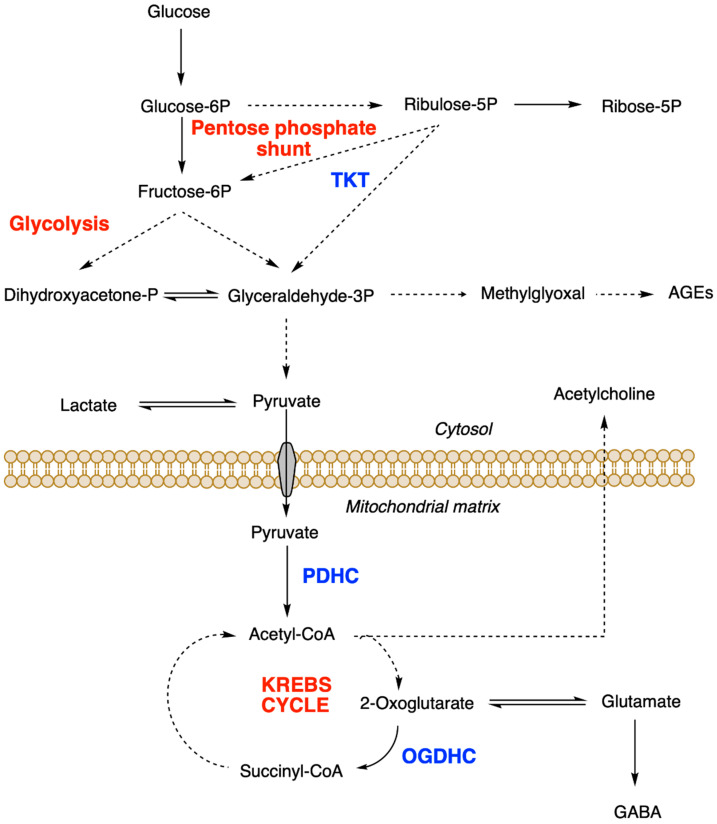
Role of ThDP-dependent enzymes in cell metabolism. TKT, transketolase; PDHC pyruvate dehydrogenase complex; OGDHC, oxoglutarate dehydrogenase complex. Broken lines represent steps involving several reactions.

In the 1930s, Peters et al. [3] investigated the biochemical mechanisms of thiamine function. For this, they used pigeons whose spastic head retraction (opisthotonus) is a characteristic sign of acute TD. They showed that addition of the vitamin was required to oxidize glucose and pyruvate in brain extracts from thiamine-deficient pigeons. It was later found that the main active form of the vitamin was not free thiamine but its diphosphorylated form thiamine pyrophosphate (or diphosphate, ThDP) [3,4]. In the following years, it was shown to act as a coenzyme for three essential enzymes or enzyme complexes: pyruvate dehydrogenase complex (PDHC), oxoglutarate dehydrogenase complex (OGDHC) and transketolase (TKT) [5]. As these enzymes catalyze essential steps in glucose oxidation, it is obvious that ThDP is an indispensable cofactor for energy metabolism (Figure 1) and it is not surprising that TD will have deleterious effects on organs that are particularly dependent on oxidative metabolism such as the nervous system and the heart. The mammalian brain is well known for its high consumption of glucose and oxygen. Moreover, the ThDP-dependent enzyme complexes PDHC and OGDHC are required for the production of important neurotransmitters such as acetylcholine, glutamate or GABA (Figure 1) [6,7]. More recent studies have shown that ThDP is a cofactor for at least three other brain enzymes (branched-chain 2-oxoacid dehydrogenase complex, 2-hydroxyacyl-CoA lyase 1 and 2-oxoadipate dehydrogenase complex), but their implications in TD disorders are less obvious [8].

These considerations have led to the widely accepted view that ThDP is the only active cellular form of the vitamin and that disorders linked to TD are the consequence of a reduced activity of ThDP-dependent enzymes in the nervous system. However, TD-disorders are complex and can adopt various forms [9]. Impairment of energy metabolism may be a satisfactory explanation for several syndromes such as some types of encephalopathies or congestive heart failure observed in wet (the cardiac form of) beriberi. However, other features of TD disorders are more difficult to explain in view of their broad clinical spectrum [10]. In the brain, TD causes memory loss, disruption of blood–brain barrier as well as inflammation (activation of glial cells) and oxidative stress [11,12,13,14].

An even more disturbing feature is the selective vulnerability of diencephalic structures in the Wernicke–Korsakoff syndrome, a TD state caused mostly by heavy drinking [15]. In Wernicke–Korsakoff’s syndrome, irreversible lesions appear in the thalamus and mammillary bodies, while the cortex is largely spared. These observations have led to the idea that thiamine (or a thiamine derivative other than ThDP) may exert neuromodulatory or neuroprotective actions through mechanisms unrelated to the coenzyme role of ThDP [16,17,18].

TD and the resulting brain disorders are rather common in humans. This vulnerability is linked to the slow carrier-mediated absorption of thiamine through the intestinal epithelium [19] and the blood–brain barrier [20]. Indeed, thiamine is a water-soluble vitamin, unable to cross biological membranes in the absence of a transport protein. Therefore, precursors with higher bioavailability have been developed to increase the absorption of the vitamin. Most of these precursors or provitamins are lipophilic compounds that freely diffuse through cell membranes. Therefore, they can easily cross the intestinal epithelium and reach the bloodstream. They are then readily converted to free thiamine and ThDP in blood and liver. Therefore, oral treatment with such precursors may result in a rapid and strong increase in blood thiamine levels.

**Figure 2:**
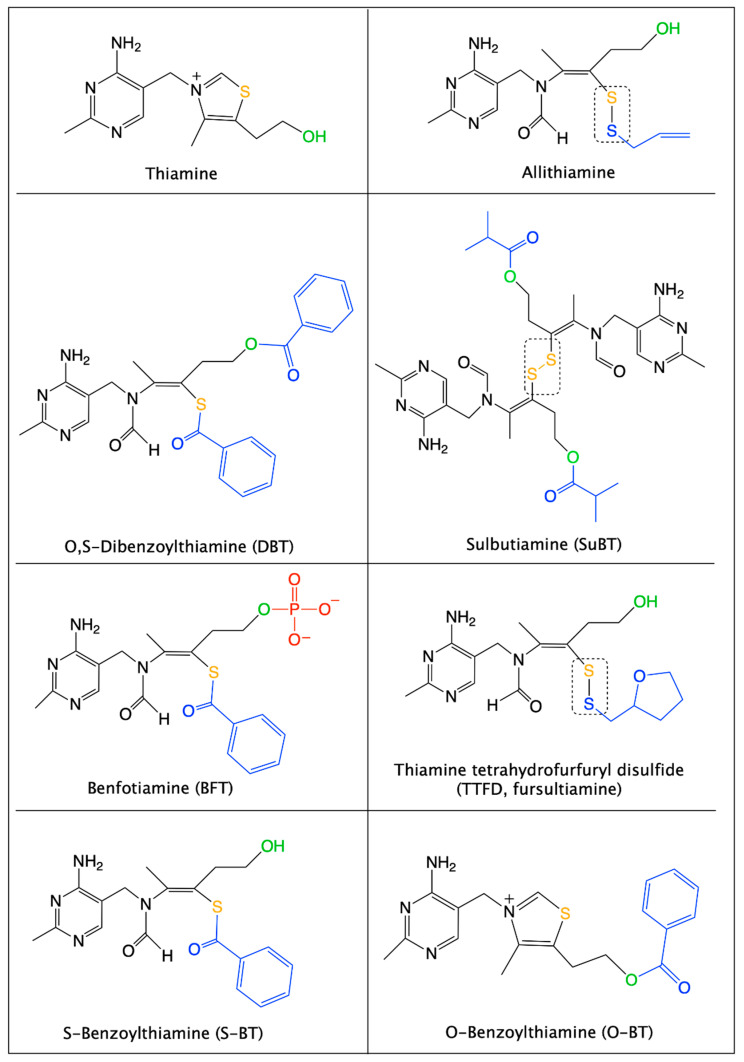
Structural formulas of thiamine and thiamine precursors with higher bioavailability. The sulfur atom of the thiamine thiazolium ring is shown in yellow. The alcohol group of thiamine is shown in green. Organic substituents are in blue and the phosphate group on BFT is shown in red. Allithiamine, TTFD and SuBT (a symmetric dimer) are disulfides (boxes), while BFT, DBT and S-BT are thioesters. O-Benzoylthiamine results from the hydrolysis of the thioester in DBT or through an intramolecular rearrangement of S-BT followed by spontaneous thiazole ring closure. Note that for all the open thiazole ring derivatives the -CH_3_ group and the sulfur group must be in “trans” position to allow thiamine to be formed. This is the case for the (Z)-isomer of BFT shown here and which is the only one that should be called “benfotiamine”.

Most of these thiamine precursors were developed in Japan in the 1950s and 1960s, the best known are allithiamine, fursultiamine (thiamine tetrahydrofurfuryl disulfide, TTFD), sulbutiamine (SuBT) and benfotiamine (BFT) (Figure 2) [21].

A most interesting and important point is that the usefulness of these compounds is not restricted to relieving the symptoms of TD. Indeed, some of them have been shown to extend beneficial effects (especially in the brain) in organisms having normal levels of thiamine and ThDP. Interesting pharmacological properties were reported for SuBT and TTFD, the former exerting antiasthenic properties [22] and the latter having some beneficial effects in a mild autism spectrum disorder [23]. Presently, the most studied precursor is BFT, which was initially shown to prevent several complications in models of experimental diabetes [24] and also to exert prominent neuroprotective effects in mouse models of neurodegeneration [25,26]. More importantly, it was recently shown that BFT treatment in humans tends to improve cognitive outcome in patients with mild Alzheimer’s disease (AD) [27,28]. In this review, we will focus on the mechanism of BFT actions in vitro and in vivo. In addition, we will also highlight the neuroprotective effects of a hitherto unexplored thiamine precursor, O,S-dibenzoylthiamine (DBT). This compound has powerful antioxidant and anti-inflammatory properties similar to BFT but is active at substantially lower concentrations [29]. Like BFT, DBT is devoid of toxic effects and may have a therapeutic potential for brain pathologies associated with oxidative stress and inflammation, i.e., neurodegenerative diseases and major depression.

## 2. Properties and Mechanism of Action of Benfotiamine

### 2.1. Structure and Physico-Chemical Properties of BFT

BFT (S-benzoylthiamine O-monophosphate, Figure 2) is an S-acyl (thioester) derivative with an open thiazole ring and a negatively charged phosphate group. Due to the presence of the negatively charged phosphate group, BFT is practically insoluble in organic solvents [30]. It is, however, soluble in water at slightly alkaline (but not acid) pH. There can be two (*E*,*Z*) isomers differing by the substitution of the double C=C bond of the thiazolium remnant. It is obvious that only the *Z* isomer can regenerate the thiazolium ring characteristic of thiamine and hence only this isomer should be called “benfotiamine”.

**Figure 3:**
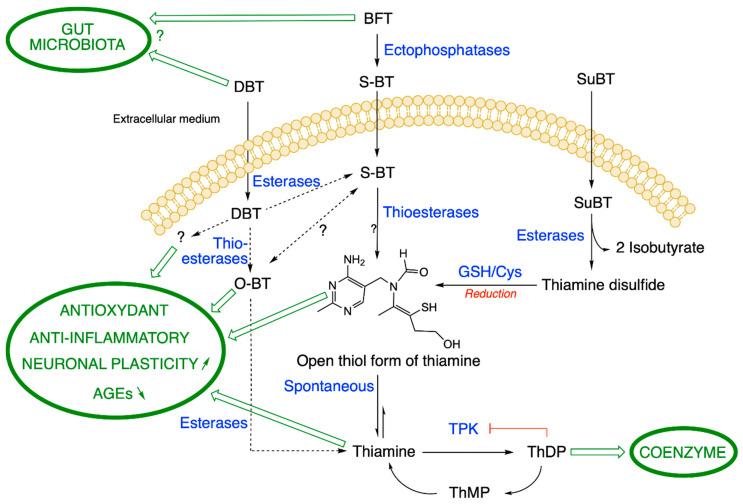
Metabolization pathways of DBT, BFT and SuBT in an ideal cell. DBT: dibenzoylthiamine; BFT: benfotiamine; S-BT: S-benzoylthiamine; SuBT: sulbutiamine; O-BT: O-benzoylthiamine; GSH: reduced glutathione; ThDP: thiamine diphosphate. Thiamine pyrophosphokinase (TPK), the enzyme responsible for ThDP synthesis is inhibited by its product ThDP by a feedback mechanism. In eukaryotes, ThMP can only be formed by hydrolysis of ThDP. As the metabolization reactions of DBT are not clear, we use broken lines. (Figure modified from [29]).

BFT differs from another group of thiamine precursors, which are disulfides (allithiamine, SuBT, TTFD) and are uncharged hydrophobic compounds. Allithiamine is the prototype of the thiamine disulfide prodrugs. D. Loew uses the term “allithiamine” to designate all thiamine prodrugs with an open thiazole ring and that are converted to thiamine after closure of this ring [30]. However, and in agreement with D. Lonsdale [31], we think that the term allithiamine should only be used for this compound, naturally formed in crushed garlic bulbs [32] or, at most, be reserved to designate the group of thiamine disulfides. In contrast, BFT and DBT are not disulfides but thioesters. This distinction is very important, because both types of compounds have different pharmacological properties [29,33] and require different pathways for metabolization (Figure 3). Indeed, disulfides require reduction (a redox reaction) by cellular thiols such as reduced glutathione (GSH) or cysteine [30], while thioesters require hydrolysis to form the open thiamine thiol form.

### 2.2. Metabolism of BFT

BFT is a fairly stable polar compound that cannot diffuse through cell membranes as it is not lipophilic (in contrast to what is claimed in many articles). However, after oral administration, BFT can be rapidly dephosphorylated in the small intestine through the action of alkaline phosphatases (ectoenzymes) bound to brush-borders of epithelial cells (Figure 3) [30]. This yields S-benzoylthiamine (S-BT) which is the lipophilic metabolite of BFT that easily diffuses through cell membranes and crosses the epithelium to reach the blood stream. Its main products in human blood are thiamine and to a much lesser extent ThMP and ThDP, mainly formed in erythrocytes [34]. Note that S-BT is not used as a thiamine precursor as it is less stable than BFT. BFT has a higher bioavailability than thiamine, thiamine disulfide or TTFD [30,35]. In humans, after a single dose, the maximum thiamine concentration was dose-proportional and was reached after 1–2 h with an elimination half-life of 6-14 h inversely depending on the dose. ThMP and ThDP have a longer half-life, because they are formed and retained in erythrocytes, while thiamine is mainly present in the plasma with the excess being eliminated by the kidneys [36]. Due to the relatively long half-life, daily administration of BFT leads to an accumulation ratio of ~2, with a steady-state being reached at day 7 [34].

However, as most analytical methods are based on the detection of fluorescent thiochrome derivative, only BFT metabolites with an intact thiazolium ring are detected [33]. Other metabolites would require the use of mass spectrometric detection [29,33,37,38]. Hence, the existence of presently unknown metabolites of BFT cannot be excluded (see also Figure 3).

The requirement for dephosphorylation explains why oral administration of BFT is more efficient than parenteral routes. However, it is not known what concentrations of S-BT can be present in blood and what could be the half-life of S-BT in this compartment. It is also unknown whether significant amounts of this compound might reach the brain parenchyma. Indeed, no thiamine precursor has ever been reported to reach the brain.

In any event, probably most of the S-BT is hydrolyzed to thiamine after a few hours through the action of thioesterases in red blood cells or in the liver. Note that the first product of hydrolysis is the open thiol form of thiamine, but this form is quite unstable at physiological pH (the equilibrium being towards the closed ring form [39]) and is spontaneously converted to thiamine in the cytosol (Figure 3). In vitro, some S-BT may also undergo a molecular rearrangement to O-benzoylthiamine (O-BT, Figure 2 and Figure 3) [40], but it is not known whether this conversion occurs in vivo.

In mice, oral treatment with BFT (100 mg/kg) strongly increases blood thiamine concentrations, the maximum being reached two hours after gavage [41]. There is also a rapid increase in thiamine and ThDP content in the liver, but not in the brain. Likewise, the brain content of thiamine derivatives remains unchanged after chronic (14 days) oral administration of BFT (100 mg/kg per day). Similar results were obtained when using mouse models of brain disorders (see below). In these studies, the mice had been fed on a thiamine-rich diet and brain ThDP content was not increased by BFT treatment. In rats, however, a recent study found a significant elevation (30–50%) in ThDP content of hippocampus and entorhinal cortex after 4-week treatment with BFT (150 mg/kg per day) [42].

We studied the metabolism of BFT in more detail in cultured neuroblastoma cells [33]. Using mass spectrometric detection and HPLC, we confirmed that BFT is unable to cross the cell membrane to a significant amount. We observed an increase in cell content of thiamine only after a lag period, when BFT was dephosphorylated to S-BT by membrane-bound phosphatases or serum phosphatases present in the culture medium. S-BT freely diffuses into the cells where it is quickly converted to thiamine, presumably through the action of intracellular thioesterases. A slower increase in cell ThDP content occurs, but only a small part of the accumulated thiamine is converted to ThDP, a reaction catalyzed by thiamine pyrophosphokinase (TPK, EC 2.7.6.2). This is because ThDP tends to block its own synthesis by a feedback mechanism (Figure 3) [43]. In eukaryotes, ThMP is only formed by hydrolysis of ThDP.

### 2.3. Overview of the Beneficial Effects of BFT Treatment in Animal Models and Humans

Like other precursors such as allithiamine, thiamine disulfide, SuBT or TTFD, BFT was first used to alleviate the symptoms of thiamine deficiency. It has long been known, for instance, that patients with type 2 diabetes are often deficient in thiamine and that a thiamine supplement is useful for the treatment of diabetic complications [44]. Thus, BFT was first used to prevent the development of complications such as diabetic neuropathy and retinopathy [24]. Other beneficial effects of BFT are the reduction of glucose toxicity in endothelial cells [45], alleviation of diabetes-induced cerebral oxidative damage [46] and rescue of cardiomyocyte contractile function in experimental diabetes [47].

In the following years, various neuroprotective effects of BFT treatment were reported not only in diabetes-induced nerve damage but in various brain pathologies, particularly in AD.

#### 2.3.1. Beneficial Effects of BFT in Mouse Models of Brain Disorders

In a landmark study, Pan et al. (2010) [25] investigated the effects of chronic treatment by BFT on amyloid precursor protein/presenilin (APP/PS1) double transgenic mice, a classic model of AD. Oral treatment (100–200 mg/kg/day) during 8 weeks had powerful beneficial effects on the animals, enhancing spatial memory as well as reducing amyloid plaque numbers and phosphorylated tau levels in cortical areas. Remarkably, these therapeutic actions were specific for BFT, and no such effects were seen when the mice were treated with TTFD or high doses of thiamine.

More recently, a similar treatment with high doses of BFT (200 mg/kg per day for 8 weeks) was applied in a mouse model of tauopathy, P301S mice [26]. In this model, BFT treatment increased lifespan, prevented death of spinal neurons and improved behavioral deficits. It also decreased oxidative stress, inflammation, accumulation of advanced glycation end products (AGEs), tau phosphorylation and formation of neurofibrillary tangles in cerebral cortex and hippocampus.

In addition to these therapeutic actions in mouse models of neurodegeneration, beneficial effects of BFT have been reported in mouse models of stress-induced anxiety, aggression and depression. Stress could be induced by a modified swim test, the presence of a predator for five consecutive nights [48,49] or chronic ultrasound exposure for 20 days [50]. In those models, BFT treatment counteracted anxiety and depression-like behavior as well as aggression linked to emotional stress. It also normalized plasticity markers, stimulated neurogenesis and improved memory. These data suggest that BFT may have a therapeutic potential not only in neurodegenerative diseases but also in other brain pathologies such as major depression linked to stressful events.

#### 2.3.2. BFT Treatment in Clinical Studies of Patients with Mild AD

Thiamine treatment does not exert significant beneficial effects in clinical trials of AD even at very high doses (3 g per day for 3–12 months) [51,52]. This was ascribed to a poor bioavailability of the vitamin and it was hoped that treatment with precursors of higher bioavailability would give better therapeutic effects. A study carried out in 1996 with TTFD (100 mg per day for 12 weeks) showed some improvement in cognitive function in mildly impaired patients [53] but no follow-up study was done. The first clinical trial of BFT treatment was conducted in 2016 on five patients with mild to moderate AD [28]. The patients, after receiving an oral dose of 300 mg of BFT per day during 18 months, had improved cognitive function independently of β-amyloid accumulation. As there were only five patients and no placebo control, these results could only be considered as preliminary, but they showed that it was worth continuing to explore this track. More recently, Gibson et al. [27] reported the results of a randomized placebo-controlled phase IIa clinical trial of a 12 month BFT treatment of patients with either mild cognitive impairment or mild to moderate AD. The participants were treated with BFT (300 mg orally twice a day) or placebo. In the blood of patients, there was a strong increase in concentrations of thiamine and ThDP, while there was a significant reduction in blood advanced glycation products (AGEs). Importantly, BFT treatment resulted in an improvement of cognitive functions of patients as assessed by several tests. The results of this pilot study are very encouraging and BFT appears to be a safe and cost-effective treatment of mild forms of AD.

In this clinical trial, as in a recent pharmacokinetic study (using doses up to 1200 mg, [34]), no adverse effects of BFT were observed, suggesting that BFT is well tolerated and safe for human use.

### 2.4. Mechanism of Action of Benfotiamine

#### 2.4.1. Effects on Glucose Metabolism and Mitochondrial Function

The mechanism underlying the cytoprotective effects of BFT was first investigated in diabetic pathologies. High glucose concentrations are believed to cause superoxide overproduction by the mitochondrial electron transport chain as well as inactivation of glyceraldehyde phosphate dehydrogenase, resulting in accumulation of glyceraldehyde 3-phosphate and increased production of methylglyoxal and AGEs (Figure 1) [54]. In cultured endothelial cells, BFT could block three major pathways of hyperglycemic damage [24]: the hexosamine pathway, the AGE formation pathway and the diacylglycerol-protein kinase C pathway. To explain those effects, the authors proposed that BFT administration causes a removal of glyceraldehyde 3-phosphate and fructose 6-phosphate through activation of TKT, a ThDP-dependent enzyme that catalyzes a rate-limiting step in the non-oxidative part of the pentose phosphate pathway (Figure 1). BFT was thus supposed to act by increasing intracellular ThDP levels. Likewise, several investigators considered that BFT might have beneficial effects in neurodegenerative diseases as it could increase brain ThDP levels, stimulating ThDP-dependent enzymes and boosting energy metabolism [55]. It is indeed well known that disturbances in glucose metabolism are associated with the pathogenesis of AD. This disease has even been considered as an “insulin-resistant brain state” [56]. On the other hand, there are several common mechanisms associated with the neurological symptoms observed in TD and AD, notably memory loss [14]. It has been known for a long time that disturbances in ThDP-dependent steps in glucose metabolism are associated with AD [57]. For instance, there is a significant decrease in OGDHC activity [58] and ThDP content [59] in post mortem brains of AD patients. More recently, it was shown that TD increases β-amyloid accumulation in the brains of AD mouse models [60].

Neuroprotective effects of BFT were tested for the first time in 2010 showing powerful beneficial effects in a mouse model of AD [25], but this occurred without any concomitant increase in brain ThDP content. Thus, the therapeutic actions of BFT do not appear to be linked to a stimulation of glucose oxidative metabolism in this model. These findings point to a specific pharmacological effect of BFT (more probably its metabolite S-BT) or a presently unsuspected thiamine derivative.

In another investigation on the neuroprotective effects of BFT in a mouse model of tauopathy, brain ThDP levels were also unchanged [26]. However, there was a significant improvement of mitochondrial function, increasing respiratory complex I immunoreactivity and superoxide dismutase activity. Interestingly, there was a marked upregulation of PGC-1α mRNA levels, suggesting that BFT treatment stimulates mitochondrial biogenesis.

Mitochondrial dysfunction is involved in the onset and progression of neurodegenerative diseases [61,62,63,64]. It is therefore likely that the beneficial effects of BFT administration on mitochondrial function is not restricted to the activation of the ThDP-dependent enzyme complexes PDHC and OGDHC. The mechanism of action of BFT metabolites on mitochondrial biogenesis and function remains largely unknown and deserves further investigation.

#### 2.4.2. Effects of BFT on Glycogen Synthase Kinase 3 (GSK3)

GSK3 is a serine-threonine protein kinase originally identified as playing an important role in glycogen metabolism. However, in the last two decades it became clear that GSK3 is a much more pleiotropic enzyme, able to phosphorylate over 100 substrates and to regulate numerous cellular functions including gene transcription, apoptosis, neurodevelopment and synaptic plasticity [65].

In mammals, there are two highly homologous isoforms of GSK3, α and β [66]. The β isoform is expressed in higher levels in the brain (particularly the hippocampus) and appears to be important for the regulation of neuronal function and plasticity [65,67,68,69].

GSK3β activity is regulated at different levels [70]. An important mechanism is its inactivation through phosphorylation on serine 9, catalyzed by the kinase AKT. This occurs when the prosurvival PI3K/AKT pathway is activated by insulin or growth factors [71,72]. Thus, neuronal survival is generally associated with downregulation of GSK3β activity.

As GSK3β touches so many aspects of cellular signaling, it is not surprising that it is also involved in a huge number of pathological processes, including psychiatric diseases and neurodegeneration. Indeed, several studies suggest that GSK3β is involved in the pathogenesis of AD [73]. An obvious reason is that GSK3β is the predominant kinase that phosphorylates tau, contributing to its hyperphosphorylation and generation of neurofibrillary tangles [74]. Moreover, it has been found that inhibition of GSK3β activity decreases the production and accumulation of amyloid-β in APP-overexpressing mice [75]. Recently, it was demonstrated that specific inhibition of GSK3β (but not of GSK3α) reduced the BACE1-mediated cleavage of APP, reducing neuritic plaque formation and alleviating memory deficits in an AD transgenic mouse model [76].

These findings are consistent with the idea that the reductions in amyloid load and tau hyperphosphorylation caused by BFT treatment in APP/PS1 mice [25] could be linked to the inhibition of GSK3α/β. These authors indeed found that BFT treatment decreased the activity of GSK3, concomitant with an elevation of the phosphorylation level of the enzyme. A BFT-induced elevation of GSK3α/β phosphorylation was also found in a rat model of AD [42].

Abnormally active GSK3 has also been linked to the pathogenesis of mood disorders. In knockin mice in which one or the other isoform of GSK3 was mutated to a hyperactive form, those with hyperactive GSK3β (but not GSK3α) displayed heightened vulnerability to the learned helplessness model of depression-like behavior [70]. Adult hippocampal neurogenesis was also severely impaired. On the other hand, changes in expression and phosphorylation of GSK3β were reported in mouse models of stress-induced anxiety and depression [48]. In the prefrontal cortex, mRNA levels of GSK3β were increased in the modified swim test and when the mice were exposed to predator stress. This increase was fully reversed when the animals were treated with BFT (200 mg/kg per day).

Taken together, these findings suggest that the beneficial effects of BFT treatment in mouse models of brain disease may involve a decrease of GSK3β activity. This decrease seems to be mostly induced by phosphorylation of GSK3β on Ser 9 by AKT, though other kinases may be involved. It should be mentioned, however, that in P301S mice (a model of tauopathy), Tapias et al. did not find alterations in phospho-GSK3β expression in brain following BFT treatment [26].

#### 2.4.3. Possible Involvement of the PI3K/AKT Pathway in Neuroprotection by BFT

The PI3K/AKT pathway is well known to promote cell growth and the AKT protein kinase appears to be a critical mediator of neuronal survival. It is thus conceivable that the cytoprotective effects of BFT may involve the stimulation of this pathway. It was indeed reported that BFT counteracts the toxic effects of high glucose in endothelial cells via AKT/FoxO signaling [45]. In APP/PS1 mice, BFT increased the phosphorylation level of AKT [25], thus increasing the activity of this kinase (that, in turn phosphorylates GSK3β).

Hence, BFT treatment can, at least to some extent, exert its neuroprotective effects through a stimulation of the PI3K/AKT pathway, ultimately resulting in inhibition of GSK3β. However, the molecular target of BFT treatment is not identified.

#### 2.4.4. Effects of BFT on the Accumulation of AGEs

Abnormal production of AGEs, a marker of impaired glucose metabolism, has been reported to occur not only in type-2 diabetes but also in neurodegenerative diseases such as AD [27,77] and mouse models of tauopathies [26]. Diabetic neuropathies and retinopathies are linked to microvascular damage. In endothelial cells, BFT (50 µM) completely prevented the accumulation of AGEs induced by high glucose [24]. This protective effect was abolished when the cells were transfected with transketolase (TKT) antisense oligonucleotides, suggesting that BFT acts through activation of TKT, a ThDP-dependent enzyme. The resulting upregulation of the pentose phosphate pathway would counteract the hyperglycemia-induced accumulation of glyceraldehyde-3 phosphate, thus reducing the production of methylglyoxal and AGEs.

In a mouse model of tauopathy BFT treatment was very effective to reduce AGE formation in brain and spinal cord [26], but the role of TKT in the protective effect is less clear. The authors reported a modest decrease of TKT activity in the brain of transgenic mice compared to control mice. There was a slight but significant increase in TKT activity in the brain of BFT-treated animals. It is not clear that such modest effects on TKT can account for the strong effects of BFT treatment on AGE accumulation. In addition, there is no evidence to show that the presence of AGEs in the transgenic mice might be linked to hyperglycemia and elevation of glyceraldehyde 3-phosphate in brain tissues.

As mentioned above (Section 2.3.2) Gibson et al. (2020) demonstrated that BFT significantly relieved the increase in AGEs in the blood of patients with mild AD during the study, but TKT activity has not been studied [27].

#### 2.4.5. Antioxidant Effects of BFT

A number of studies have suggested that thiamine and its derivatives may protect the brain from oxidative damage. Indeed, oxidative stress is known to be associated with TD [11,78]. On the other hand, it is well known that the brain is particularly vulnerable to oxidative stress via its high oxygen consumption, and oxidative damage increases in the aging brain, especially if neurodegeneration occurs. It was thus appealing to consider that the beneficial effects of treatments with thiamine precursors in nerve and brain pathologies may be linked, at least in part, to antioxidant effects.

An early publication suggested that BFT alleviates diabetes-induced cerebral oxidative damage, independently of AGEs [46]. A more recent study showed that, in a mouse model of anxiety and depression caused by predation stress, there is an increase of protein carbonylation (a marker of oxidative stress) in the hippocampus of stressed mice. This increase is fully reversed by treatment with BFT or high doses of thiamine [49]. Similar results were reported with mice submitted to chronic ultrasound exposure [50].

In a transgenic mouse model of tauopathy, oxidative damage (lipid peroxidation) in spinal cord sections is reversed by BFT (200 mg/mg per day) [26]. This treatment also increases the expression of thioredoxin and some oxidative stress-protective enzymes known to be under the control of the transcription factor Nrf2. Under normal conditions, Nrf2 remains sequestered in the cytoplasm through its binding to Keap1. Reactive oxygen species (ROS) or electrophilic compounds can react with Keap1 and dissociate it from Nrf2, which can then diffuse to the nucleus, where it can bind to a promoter element called the antioxidant response element (ARE). This activates the expression of a battery of genes involved in protection against oxidative stress [79].

Tapias et al. [26] proposed a model in which putative products of BFT metabolism such as S-BT and O-BT (Figure 1) would react with Keap1 and allow Nrf2 to enter the nucleus and activate the expression of antioxidant genes. They indeed showed that, in embryonic fibroblasts, BFT, S-BT and O-BT could stimulate the expression of Nrf2-dependent genes, but only at very high concentrations (100 µM). It seems very unlikely that such high concentrations of BFT metabolites can be reached in the brain parenchyma. In any event, it should be recalled that, so far, no metabolite of BFT other than thiamine could be detected in the blood, let alone in the brain.

Antioxidant effects of BFT were also studied in vitro. In cultured neuroblastoma cells, BFT (25–50 µM) protected the cells from paraquat-induced cell death [33]. Paraquat is known to be toxic to many cell types, including neurons, through the production of ROS, particularly superoxide anions. Paraquat increases protein carbonylation in mouse neuroblastoma cells and this was relieved by BFT. The protective effects of BFT seem to be linked to the accumulation of high concentrations of thiamine rather than the coenzyme ThDP in the cells. However, the protection against ROS toxicity was not due to a direct interaction of thiamine with ROS. It is thus likely that the protective effects of BFT are linked to an indirect mechanism.

An obvious possibility is that the accumulation of intracellular thiamine might activate the Nrf2/ARE pathway. However, this pathway was only weakly activated by BFT treatment. This is not surprising as thiamine has only poor electrophilic properties and should not easily react with Keap1 [26]. It could be argued that protection of the cells may be due to the presence of intracellular S-BT, this metabolite of BFT being more likely to react with Keap1 [26]. However, there is little accumulation of S-BT in the cytoplasm, as it is quickly converted to thiamine by thioesterases. In addition, SuBT, a disulfide precursor of thiamine which cannot be converted to S-BT (Figure 2) is practically as effective as BFT to protect the cells from paraquat toxicity. These data are strong evidence that, in cultured neuroblastoma cells, the active metabolite of BFT is thiamine, although it cannot be excluded that some unknown metabolite of thiamine might be more active. Those in vitro studies also strongly suggest that the stimulation of the Nrf2/ARE pathway is not sufficient to explain all the antioxidant effects of BFT. Hence, alternative mechanisms should be explored. As mentioned above, BFT treatment can improve mitochondrial biogenesis and function (increasing the activity of superoxide dismutase) but the underlying mechanisms are unknown. On the other hand, in BV2 microglial cells, BFT (50–250 µM) markedly increases the glutathione content of the cells [80]. This suggests that enzymes of glutathione metabolism are possible targets for the antioxidant actions of BFT. Microglial cells are resident macrophage-like immune cells of the nervous system. Overactivation of microglia induces the production of neurotoxic reactive oxygen and nitrogen species. This process is likely to play an important role in neurodegenerative processes [81].

#### 2.4.6. Anti-Inflammatory Effects of BFT

According to earlier studies (see [82]), TD is associated not only with oxidative stress in brain but also with inflammatory processes such as microglial activation [83]. This raises the possibility that thiamine precursors may exert anti-inflammatory actions. Indeed, BFT treatment relieves inflammatory pain in rats [84]. P301S mice (a model of tauopathy) have increased immunoreactivity of iNOS, COX-2, TNF-α and IL-1β in spinal cord neurons and this was counteracted by chronic treatment with BFT [26].

The potential mechanism of the anti-inflammatory effects of BFT was studied in vitro. In human macrophages treatment with lipopolysaccharide (LPS), a well-known inducer of inflammation in these cells, caused the appearance of cytotoxic signals [85]. This response was significantly suppressed in the presence of 100 µM BFT. BFT downregulated proapoptotic signals and decreased the production of inflammatory marker proteins such as iNOS and COX-2. Further, phosphorylation and degradation of IκB and consequent activation and nuclear translocation of the transcription factor NF-κB were significantly prevented by BFT.

In basal conditions, NF-κB is sequestered in the cytoplasm through binding to IκB. When the inflammatory process is induced (e.g., by LPS), NF-κB separates from IκB and can diffuse to the nucleus where it induces the expression of proinflammatory genes such as iNOS and TNF-α. IκB is thus a possible target for an active metabolite of BFT.

BFT counteracts the morphological changes corresponding to the LPS-induced activation of the microglial cells. In addition, it decreases the production of proinflammatory mediators such as iNOS, COX-2, TNF-α and IL6. These effects of BFT are likely mediated by suppression of NF-κB translocation to the nucleus. BFT also suppresses phosphorylation of the kinases ERK1/2, JNK and AKT [86]. Another study suggested that BFT inhibits the release of proinflammatory metabolites of arachidonic acid in macrophages [87].

However, all these effects require rather high concentrations (50–250 µM) of BFT and they may thus not be relevant in vivo. Notwithstanding, thiamine administration in high doses clearly has anti-inflammatory properties in vivo [88].

#### 2.4.7. Effects of BFT on Glutamate Receptors, Synaptic Plasticity and Neurogenesis

Several studies have shown that BFT treatment can improve learning and memory in mouse models of brain pathologies. This was the case in APP/PS1 mice, a model of AD [25] and in P301S mice, a model of tauopathy [26]. These observations led to consider the possibility that BFT treatment might increase the expression of NMDA receptors in the brain. These receptors indeed play an essential role in memory formation in the hippocampus and are altered in many brain pathologies including neurodegenerative diseases and depression [89].

BFT treatment increases cognitive abilities in rats treated by intracerebroventricular injections of streptozotocin, a model of AD. There was a simultaneous increase in the expression of the NMDA-type-glutamate receptor subunit 2B (GluN2B) in hippocampus and entorhinal cortex [42].

AMPA-type glutamate receptors are tetramers composed of subunits GluA1-4. They mediate fast synaptic transmission involved in neuroplastic processes. In mice exposed to chronic ultrasound exposure (resulting in aggressive behavior), Gorlova et al. reported altered AMPA receptors subunits expression and decreased expression of plasticity markers PSD95, PSA-NCAM and β-catenin [50]. The administration of thiamine or BFT (200 mg/kg per day) decreased aggression, reversed ultrasound-induced changes in GluA1 and GluA2 subunit expression and reversed the decreased expression of plasticity markers.

The effect of BFT treatment on neurogenesis was studied in mice exposed to predator stress for five consecutive nights. In this model, there is a marked, stress-induced, decrease of proliferation (number of Ki67-positive cells) and survival (number of BrdU-positive cells) of newborn immature neurons in the subgranular zone of the dentate gyrus. These reductions were counteracted by treatment with thiamine or BFT, the latter being the most effective [49].

## 3. Neuroprotective Properties of Dibenzoylthiamine

Though powerful beneficial effects of BFT treatment have been reported in mouse models of neurodegeneration and other brain pathologies, this requires the administration of high doses (100–200 mg/kg per day). If comparable, this would correspond to about 10 g per day in humans. It may seem surprising, therefore, that a daily dose of 600 mg was effective to improve cognitive function in patients with moderate AD (see Section 2.3.2, [27]). Though BFT has no significant side effects, it seems difficult to administer much higher doses of BFT to patients. Therefore, there is a need to find precursors having beneficial effects similar to BFT but acting at lower doses.

Recently, it was found that a hitherto unexplored derivative, DBT (Figure 1) could meet these requirements [29]. DBT has actually been known for a long time and is allowed as a food additive in Japan. No toxic or tumorigenic side effects have been reported [90], yet there are practically no data about its biological effects. In salmon yearlings, it was better tolerated than thiamine or BFT and it also led to a higher retention of thiamine over time [91].

DBT is composed of a thiamine molecule with an open thiazolium ring linked to two molecules of benzoate, one via a thioester the other via an O-ester bond. Hence, its conversion to thiamine requires the action of two different enzymes: a thioesterase and an esterase (Figure 3). DBT is lipophilic, yet it can be dissolved in water at slightly acid pH. It strongly increases the intracellular thiamine concentrations, but there is almost no formation of S-BT, one of the main degradation products of BFT. DBT (10 mg/kg, intraperitoneal injection) increased the level of thiamine and ThDP in the blood of mice. The maximum levels were obtained after two hours. However, no significant increase of ThDP level was observed in the liver or the brain of the animals [29].

In vitro studies showed that low (5–10 µM) concentrations of DBT protect neuroblastoma cells from paraquat toxicity by counteracting oxidative stress [29]. The concentrations of BFT necessary to obtain the same protection were 5–10-fold higher (see Section 2.4.5). Concerning the mechanisms involved, it was found that DBT increases the synthesis of reduced glutathione and NADPH in a Nrf2-independent manner. As for BFT, these effects also seem to be independent of the coenzyme role of ThDP.

DBT is also more effective as an anti-inflammatory agent than BFT. DBT (10–50 µM) protects BV2 cells from LPS-induced inflammatory processes (increased expression of iNOS and TNF-α and production of nitric oxide) by suppressing the translocation of NF-κB into the nucleus. The same types of anti-inflammatory effects were observed with BFT (50 µM) but they were clearly weaker.

Finally, chronic administration of DBT (30 mg/kg per day) arrested motor dysfunction in FUS transgenic mice, a model of amyotrophic lateral sclerosis, and relieved depressive-like behavior in mice (25 mg/kg per day) submitted to chronic ultrasound stress [29].

Taken together, those data suggest that DBT has a therapeutic potential in brain pathologies associated with oxidative stress and inflammation, not only in neurodegenerative diseases, but also major depression and aggression linked to stressful events.

## 4. Conclusions and Perspectives

During the last 20 years, a number of studies have shown that BFT has powerful beneficial effects in animal models and brain pathologies, i.e., neurodegenerative diseases and stress-induced anxiety, aggression and depression. No toxic side-effects of this compound have been reported. Recently, BFT treatment during 12 months resulted in improved cognitive functions in patients with mild to moderate AD [27]. Thus, BFT may be considered as a potentially safe and cost-effective treatment for a number of brain diseases.

In spite of positive points, the therapeutic potential of BFT remains limited as, in animal models, full beneficial actions require the administration of high doses (100–200 mg/kg per day) for long periods. Since such high doses can hardly be used in humans, it is desirable to develop more potent compounds with similar beneficial actions.

Recently, a very potent thiamine precursor, DBT, exerted antioxidant and anti-inflammatory effects in vitro and in vivo at doses substantially lower than those required with BFT. Like BFT, the compound appears to be devoid of toxic side effects. However, it remains to be checked whether a prolonged use (e.g., as a therapy for AD patients for instance) would be completely safe.

A very recent study showed a lowering of the Th-17 cell-mediated IL-17 response by thiamine (200 mg per day for three weeks) in patients with elevated proinflammatory cytokines and suggested the use of this vitamin to target the COVID-19 cytokine storm observed in some patients [88]. We expect that DBT, because of its much higher anti-inflammatory properties compared to thiamine [29], would be even more efficient.

The possibility of synergistic effects of thiamine with other validated drugs should also be considered. For instance, a recent study found that coadministration of citicoline (cytidine diphosphate-choline, an intermediate in the synthesis of phosphatidylcholine) and BFT was more efficient than each compound alone in improving memory in the passive avoidance test in a mouse model of streptozotocin-induced memory impairment [92].

A problem concerning the use of BFT and DBT is that, so far, their mechanisms of action are not well understood. It has become rather obvious that these compounds do not only act as thiamine precursors, i.e., by increasing blood thiamine concentrations or brain ThDP levels. Indeed, they exert specific pharmacological effects that are not mimicked by the administration of high doses of thiamine or even SuBT (which is not a thioester but a disulfide that requires reduction, Figure 3) [22,29,33]. A recapitulation of the properties and the effects of BFT and DBT is shown in Table 1. It is therefore likely that unidentified metabolites are formed in blood or liver (possibly an open thiol form of thiamine, Figure 3) and that some of them can reach the central nervous system. In any event, the concentrations of these active derivatives in the brain are probably very low and, consequently, they would be very potent neuroprotective agents. It is therefore of primary importance to identify these agents.

As BFT and DBT are administered orally, an alternative hypothesis might be an action on the gut microbiota, by increasing the availability of thiamine to microorganisms in an environment with, otherwise, limited resources [93,94]. Indeed, a lot of recent research tends to show that the gut microbiota are implicated in the etiopathogenesis or manifestation of many neurodevelopmental, psychiatric and neurodegenerative diseases [95].

This leads to the conclusion that BFT and DBT have pleiotropic effects that can be attributed to at least three modes of action: they strongly increase circulating thiamine concentrations, they probably produce other specific metabolites or metabolites of thiamine and they may favor growth of beneficial microorganisms of the gut microbiota.

The identification of the active derivatives of BFT and DBT and the clarification of their mechanisms of action would open new avenues in the field of neuroprotection, with the development of very potent and safe compounds for treating neurodegenerative and other brain diseases.

## Figures and Tables

**Table 1:** Recapitulation of the properties and pharmacological effects of BFT and DBT.

Properties	BFT	DBT
**Physicochemical properties**		
Solubility in organic solvents	No	Yes
Solubility in aqueous solutions	Yes (pH > 8)	Yes (pH < 6)
Metabolization		
Enzymes [29,33]	Ectophosphatases/thioesterases	Estererases/thioesterases
Main metabolites [29,33]	Thiamine, S-BT	Thiamine, O-BT (?)
**Pharmacological effects**		
Antioxidant effects (Nrf2-independent)	Yes (≤50 µM) [33]	Yes (≤50 µM) (↑ GSH and NADPH) [29]
Anti-inflammatory effects (probably via NF-κB)	↓ iNOS and TNF-α [29,86]	↓ iNOS and TNF-α [29]
Anti-AGEs effects	↓ In blood of AD patients [27]	Not tested
Neuroprotective effects in neurodegenerative diseases and models	Slows down cognitive decline in AD patients [27,28] Decreases β-amyloid load and tauopathy in mouse model of AD [25]	Not tested in AD, but arrests motor dysfunction in a mouse model of amyotrophic lateral sclerosis, and relieves depressive-like behavior in mice submitted to chronic ultrasound stress
Effects on neuronal plasticity	↑ NMDAR AMPAR expression ^1^ ↑ Neurogenesis ^2^	Not tested
**Possible molecular targets**		
TKT	Possibly increased activity [24]	No effect [29]
GSK3β	Inhibition by phosphorylation [25,42] Reduced expression [48]	Not tested
PI3K/AKT pathway	Activation [46]	Not tested
Nrf-2	At concentrations > 100 µM [26]	Not tested
NF-κB	Inhibits LPS-induced nuclear translocation [29,86]	Inhibits LPS-induced nuclear translocation [29]

^1^ After stress-induced decrease in mouse models [42,50]; ^2^ after predator stress-induced suppression [49].

## Data Availability

Not applicable.

## Abbreviations

AD	Alzheimer’s disease
AGE(s)	advanced glycation end product(s)
APP	amyloid precursor protein
DBT	dibenzoylthiamine
GSH	reduced glutathione
GSK	glycogen synthase kinase
LPS	lipopolysaccharide
O-BT	O-benzoylthiamine
ROS	reactive oxygen species
OGDHC	2-oxoglutarate dehydrogenase complex
PDHC	pyruvate dehydrogenase complex
S-BT	S-benzoylthiamine
SuBT	sulbutiamine
TD	thiamine deficiency
ThDP	thiamine diphosphate
ThMP	thiamine monophosphate
TKT	transketolase
TPK	thiamine pyrophosphokinase
TTFD	thiamine tetrahydrofurfuryl disulfide

## References

[1] Jansen B.C.P., Donath W.F. (1926). On the Isolation of Anti-Beriberi Vitamin. Proc. Kon. Ned. Akad. Wet..

[2] Grijns G. (1901). Over Polyneuritis Gallinarum. I. Geneesk Tijdscht Ned. Ind..

[3] Peters R.A. (1936). The Biochemical Lesion in Vitamin B1 Deficiency. Application of Modern Biochemical Analysis in Its Diagnosis. Lancet.

[4] Lohmann K., Schuster P. (1937). Untersuchungen Über Die Cocarboxylase. Biochem. Z..

[5] McCandless D.W. (2010). Thiamine Deficiency and Associated Clinical Disorders. Contemporary Clinical Neuroscience.

[6] Page M.G., Ankoma-Sey V., Coulson W.F., Bender D.A. (1989). Brain Glutamate and Gamma-Aminobutyrate (GABA) Metabolism in Thiamin- Deficient Rats. Br. J. Nutr..

[7] Plaitakis A., Hwang E.C., Woert M.H., Szilagyi P.E., Berl S. (1982). Effect of Thiamin Deficiency on Brain Neurotransmitter Systems. Ann. N. Y. Acad. Sci..

[8] Bettendorff L., Marriott B., Birt D.F., Stalling V., Yates A. (2020). Basic Nutrition and Metabolism. Present Knowledge in Nutrition.

[9] Whitfield K.C., Bourassa M.W., Adamolekun B., Bergeron G., Bettendorff L., Brown K.H., Cox L., Fattal-Valevski A., Fischer P.R., Frank E.L. (2018). Thiamine Deficiency Disorders: Diagnosis, Prevalence, and a Roadmap for Global Control Programs. Ann N. Y. Acad. Sci..

[10] Smith T.J., Johnson C.R., Koshy R., Hess S.Y., Qureshi U.A., Mynak M.L., Fischer P.R. (2020). Thiamine Deficiency Disorders: A Clinical Perspective. Ann. N. Y. Acad. Sci..

[11] Calingasan N.Y., Chun W.J., Park L.C., Uchida K., Gibson G.E. (1999). Oxidative Stress Is Associated with Region-Specific Neuronal Death during Thiamine Deficiency. J. Neuropathol. Exp. Neurol..

[12] Karuppagounder S.S., Shi Q., Xu H., Gibson G.E. (2007). Changes in Inflammatory Processes Associated with Selective Vulnerability Following Mild Impairment of Oxidative Metabolism. Neurobiol. Dis..

[13] Hazell A.S., Faim S., Wertheimer G., Silva V.R., Marques C.S. (2013). The Impact of Oxidative Stress in Thiamine Deficiency: A Multifactorial Targeting Issue. Neurochem. Int..

[14] Gibson G.E., Hirsch J.A., Fonzetti P., Jordan B.D., Cirio R.T., Elder J. (2016). Vitamin B1 (Thiamine) and Dementia. Ann. N. Y. Acad. Sci..

[15] Chandrakumar A., Bhardwaj A., W’t Jong G. (2018). Review of Thiamine Deficiency Disorders: Wernicke Encephalopathy and Korsakoff Psychosis. J. Basic Clin. Physiol. Pharmacol..

[16] Bettendorff L. (1994). Thiamine in Excitable Tissues: Reflections on a Non-Cofactor Role. Metab. Brain Dis..

[17] Bettendorff L., Wins P. (2009). Thiamin Diphosphate in Biological Chemistry: New Aspects of Thiamin Metabolism, Especially Triphosphate Derivatives Acting Other than as Cofactors. FEBS J..

[18] Aleshin V.A., Mkrtchyan G.V., Bunik V.I. (2019). Mechanisms of Non-Coenzyme Action of Thiamine: Protein Targets and Medical Significance. Biochem. Biokhimiia.

[19] Said H.M. (2011). Intestinal Absorption of Water-Soluble Vitamins in Health and Disease. Biochem. J..

[20] Greenwood J., Love E.R., Pratt O.E. (1982). Kinetics of Thiamine Transport across the Blood-Brain Barrier in the Rat. J. Physiol..

[21] Lonsdale D. (2006). A Review of the Biochemistry, Metabolism and Clinical Benefits of Thiamin(e) and Its Derivatives. Evid. Based Complementary Altern. Med..

[22] Starling-Soares B., Carrera-Bastos P., Bettendorff L. (2020). Role of the Synthetic B1 Vitamin Sulbutiamine on Health. J. Nutr. Metab..

[23] Lonsdale D., Shamberger R.J., Audhya T. (2002). Treatment of Autism Spectrum Children with Thiamine Tetrahydrofurfuryl Disulfide: A Pilot Study. Neuroendocr. Lett..

[24] Hammes H.P., Du X., Edelstein D., Taguchi T., Matsumura T., Ju Q., Lin J., Bierhaus A., Nawroth P., Hannak D. (2003). Benfotiamine Blocks Three Major Pathways of Hyperglycemic Damage and Prevents Experimental Diabetic Retinopathy. Nat. Med..

[25] Pan X., Gong N., Zhao J., Yu Z., Gu F., Chen J., Sun X., Zhao L., Yu M., Xu Z. (2010). Powerful Beneficial Effects of Benfotiamine on Cognitive Impairment and Beta-Amyloid Deposition in Amyloid Precursor Protein/Presenilin-1 Transgenic Mice. Brain.

[26] Tapias V., Jainuddin S., Ahuja M., Stack C., Elipenahli C., Vignisse J., Gerges M., Starkova N., Xu H., Starkov A.A. (2018). Benfotiamine Treatment Activates the Nrf2/ARE Pathway and Is Neuroprotective in a Transgenic Mouse Model of Tauopathy. Hum. Mol. Genet..

[27] Gibson G.E., Luchsinger J.A., Cirio R., Chen H., Franchino-Elder J., Hirsch J.A., Bettendorff L., Chen Z., Flowers S., Gerber L. (2020). Benfotiamine and Cognitive Decline in Alzheimer’s Disease: Results of a Randomized Placebo-Controlled Phase IIa Clinical Trial. J. Alzheimers Dis. JAD.

[28] Pan X., Chen Z., Fei G., Pan S., Bao W., Ren S., Guan Y., Zhong C. (2016). Long-Term Cognitive Improvement after Benfotiamine Administration in Patients with Alzheimer’s Disease. Neurosci. Bull..

[29] Sambon M., Gorlova A., Demelenne A., Alhama-Riba J., Coumans B., Lakaye B., Wins P., Fillet M., Anthony D.C., Strekalova T. (2020). Dibenzoylthiamine Has Powerful Antioxidant and Anti-Inflammatory Properties in Cultured Cells and in Mouse Models of Stress and Neurodegeneration. Biomedicines.

[30] Loew D. (1996). Pharmacokinetics of Thiamine Derivatives Especially of Benfotiamine. Int. J. Clin. Pharmacol. Ther..

[31] Lonsdale D. (2004). Benfotiamine and Allithiamine Should Be Differentiated. Townsend Lett. Dr. Patients.

[32] Fujiwara M., Watanabe H., Katsui K. (1954). Allithiamine, a Newly Found Derivative of Vitamin B1. J. Biochem..

[33] Sambon M., Napp A., Demelenne A., Vignisse J., Wins P., Fillet M., Bettendorff L. (2019). Thiamine and Benfotiamine Protect Neuroblastoma Cells against Paraquat and β-Amyloid Toxicity by a Coenzyme-Independent Mechanism. Heliyon.

[34] Sheng L., Cao W., Lin P., Chen W., Xu H., Zhong C., Yuan F., Chen H., Li H., Liu C. (2021). Safety, Tolerability and Pharmacokinetics of Single and Multiple Ascending Doses of Benfotiamine in Healthy Subjects. Drug Des. Devel. Ther..

[35] Bitsch R., Wolf M., Moller J., Heuzeroth L., Gruneklee D. (1991). Bioavailability Assessment of the Lipophilic Benfotiamine as Compared to a Water-Soluble Thiamin Derivative. Ann. Nutr. Metab..

[36] Gangolf M., Czerniecki J., Radermecker M., Detry O., Nisolle M., Jouan C., Martin D., Chantraine F., Lakaye B., Wins P. (2010). Thiamine Status in Humans and Content of Phosphorylated Thiamine Derivatives in Biopsies and Cultured Cells. PLoS ONE.

[37] Kim J., Jonus H.C., Zastre J.A., Bartlett M.G. (2019). Development of an IPRP-LC-MS/MS Method to Determine the Fate of Intracellular Thiamine in Cancer Cells. J. Chromatogr. B Analyt. Technol. Biomed. Life. Sci..

[38] Jonus H.C., Byrnes C.C., Kim J., Valle M.L., Bartlett M.G., Said H.M., Zastre J.A. (2020). Thiamine Mimetics Sulbutiamine and Benfotiamine as a Nutraceutical Approach to Anticancer Therapy. Biomed. Pharm. Biomed. Pharm..

[39] Duclos J.M., Haake P. (1974). Ring Opening of Thiamine Analogs. The Role of Ring Opening in Physiological Function. Biochemistry.

[40] Hurt J.K., Coleman J.L., Fitzpatrick B.J., Taylor-Blake B., Bridges A.S., Vihko P., Zylka M.J. (2012). Prostatic Acid Phosphatase Is Required for the Antinociceptive Effects of Thiamine and Benfotiamine. PLoS ONE.

[41] Volvert M.L., Seyen S., Piette M., Evrard B., Gangolf M., Plumier J.C., Bettendorff L. (2008). Benfotiamine, a Synthetic S-Acyl Thiamine Derivative, Has Different Mechanisms of Action and a Different Pharmacological Profile than Lipid-Soluble Thiamine Disulfide Derivatives. BMC Pharmacol..

[42] de Moraes R.C.M., Singulani M.P., de Gonçalves A.C., Portari G.V., da Silva Torrão A. (2020). Oral Benfotiamine Reverts Cognitive Deficit and Increase Thiamine Diphosphate Levels in the Brain of a Rat Model of Neurodegeneration. Exp. Gerontol..

[43] Voskoboyev A.I., Ostrovsky Y.M. (1982). Thiamin Pyrophosphokinase: Structure, Properties, and Role in Thiamin Metabolism. Ann. N. Y. Acad. Sci..

[44] Thornalley P.J. (2005). The Potential Role of Thiamine (Vitamin B1) in Diabetic Complications. Curr. Diabetes Rev..

[45] Marchetti V., Menghini R., Rizza S., Vivanti A., Feccia T., Lauro D., Fukamizu A., Lauro R., Federici M. (2006). Benfotiamine Counteracts Glucose Toxicity Effects on Endothelial Progenitor Cell Differentiation via Akt/FoxO Signaling. Diabetes.

[46] Wu S., Ren J. (2006). Benfotiamine Alleviates Diabetes-Induced Cerebral Oxidative Damage Independent of Advanced Glycation End-Product, Tissue Factor and TNF-Alpha. Neurosci. Lett..

[47] Ceylan-Isik A.F., Wu S., Li Q., Li S.Y., Ren J. (2006). High-Dose Benfotiamine Rescues Cardiomyocyte Contractile Dysfunction in Streptozotocin-Induced Diabetes Mellitus. J. Appl. Physiol..

[48] Markova N., Bazhenova N., Anthony D.C., Vignisse J., Svistunov A., Lesch K.-P., Bettendorff L., Strekalova T. (2017). Thiamine and Benfotiamine Improve Cognition and Ameliorate GSK-3β-Associated Stress-Induced Behaviours in Mice. Prog. Neuropsychopharmacol. Biol. Psychiatry.

[49] Vignisse J., Sambon M., Gorlova A., Pavlov D., Caron N., Malgrange B., Shevtsova E., Svistunov A., Anthony D.C., Markova N. (2017). Thiamine and Benfotiamine Prevent Stress-Induced Suppression of Hippocampal Neurogenesis in Mice Exposed to Predation without Affecting Brain Thiamine Diphosphate Levels. Mol. Cell. Neurosci..

[50] Gorlova A., Pavlov D., Anthony D.C., Ponomarev E., Sambon M., Proshin A., Shafarevich I., Babaevskaya D., Lesch K.-P., Bettendorff L. (2019). Thiamine and Benfotiamine Counteract Ultrasound-Induced Aggression, Normalize AMPA Receptor Expression and Plasticity Markers, and Reduce Oxidative Stress in Mice. Neuropharmacology.

[51] Nolan K.A., Black R.S., Sheu K.F., Langberg J., Blass J.P. (1991). A Trial of Thiamine in Alzheimer’s Disease. Arch. Neurol..

[52] Blass J.P., Gleason P., Brush D., DiPonte P., Thaler H. (1988). Thiamine and Alzheimer’s Disease. A Pilot Study. Arch. Neurol..

[53] Mimori Y., Katsuoka H., Nakamura S. (1996). Thiamine Therapy in Alzheimer’s Disease. Metab. Brain Dis..

[54] Brownlee M. (2005). The Pathobiology of Diabetic Complications: A Unifying Mechanism. Diabetes.

[55] Gibson G.E., Blass J.P. (2007). Thiamine-Dependent Processes and Treatment Strategies in Neurodegeneration. Antioxid. Redox Signal..

[56] Steen E., Terry B.M., Rivera E.J., Cannon J.L., Neely T.R., Tavares R., Xu X.J., Wands J.R., de la Monte S.M. (2005). Impaired Insulin and Insulin-like Growth Factor Expression and Signaling Mechanisms in Alzheimer’s Disease--Is This Type 3 Diabetes?. J. Alzheimers Dis. JAD.

[57] Butterworth R.F., Besnard A.M. (1990). Thiamine-Dependent Enzyme Changes in Temporal Cortex of Patients with Alzheimer’s Disease. Metab. Brain Dis..

[58] Mastrogiacomo F., Bergeron C., Kish S.J. (1993). Brain Alpha-Ketoglutarate Dehydrogenase Complex Activity in Alzheimer’s Disease. J. Neurochem..

[59] Mastrogiacomo F., Bettendorff L., Grisar T., Kish S.J. (1996). Brain Thiamine, Its Phosphate Esters, and Its Metabolizing Enzymes in Alzheimer’s Disease. Ann. Neurol..

[60] Karuppagounder S.S., Xu H., Shi Q., Chen L.H., Pedrini S., Pechman D., Baker H., Beal M.F., Gandy S.E., Gibson G.E. (2009). Thiamine Deficiency Induces Oxidative Stress and Exacerbates the Plaque Pathology in Alzheimer’s Mouse Model. Neurobiol. Aging.

[61] Lin M.T., Beal M.F. (2006). Mitochondrial Dysfunction and Oxidative Stress in Neurodegenerative Diseases. Nature.

[62] Johri A., Beal M.F. (2012). Mitochondrial Dysfunction in Neurodegenerative Diseases. J. Pharmacol. Exp. Ther..

[63] Liang W.S., Reiman E.M., Valla J., Dunckley T., Beach T.G., Grover A., Niedzielko T.L., Schneider L.E., Mastroeni D., Caselli R. (2008). Alzheimer’s Disease Is Associated with Reduced Expression of Energy Metabolism Genes in Posterior Cingulate Neurons. Proc. Natl. Acad. Sci. USA.

[64] Lee T., Lee H. (2021). Shared Blood Transcriptomic Signatures between Alzheimer’s Disease and Diabetes Mellitus. Biomedicines.

[65] Beurel E., Grieco S.F., Jope R.S. (2015). Glycogen Synthase Kinase-3 (GSK3): Regulation, Actions, and Diseases. Pharmacol. Ther..

[66] Kaidanovich-Beilin O., Woodgett J.R. (2011). GSK-3: Functional Insights from Cell Biology and Animal Models. Front. Mol. Neurosci..

[67] Woodgett J.R. (1990). Molecular Cloning and Expression of Glycogen Synthase Kinase-3/Factor A. EMBO J..

[68] Perez-Costas E., Gandy J.C., Melendez-Ferro M., Roberts R.C., Bijur G.N. (2010). Light and Electron Microscopy Study of Glycogen Synthase Kinase-3beta in the Mouse Brain. PLoS ONE.

[69] Peineau S., Taghibiglou C., Bradley C., Wong T.P., Liu L., Lu J., Lo E., Wu D., Saule E., Bouschet T. (2007). LTP Inhibits LTD in the Hippocampus via Regulation of GSK3beta. Neuron.

[70] Pardo M., Abrial E., Jope R.S., Beurel E. (2016). GSK3β Isoform-Selective Regulation of Depression, Memory and Hippocampal Cell Proliferation. Genes Brain Behav..

[71] Cross D.A., Alessi D.R., Cohen P., Andjelkovich M., Hemmings B.A. (1995). Inhibition of Glycogen Synthase Kinase-3 by Insulin Mediated by Protein Kinase B. Nature.

[72] Beaulieu J.-M., Gainetdinov R.R., Caron M.G. (2009). Akt/GSK3 Signaling in the Action of Psychotropic Drugs. Annu. Rev. Pharmacol. Toxicol..

[73] Hooper C., Killick R., Lovestone S. (2008). The GSK3 Hypothesis of Alzheimer’s Disease. J. Neurochem..

[74] Takashima A. (2006). GSK-3 Is Essential in the Pathogenesis of Alzheimer’s Disease. J. Alzheimers Dis..

[75] Rockenstein E., Torrance M., Adame A., Mante M., Bar-on P., Rose J.B., Crews L., Masliah E. (2007). Neuroprotective Effects of Regulators of the Glycogen Synthase Kinase-3beta Signaling Pathway in a Transgenic Model of Alzheimer’s Disease Are Associated with Reduced Amyloid Precursor Protein Phosphorylation. J. Neurosci. Off. J. Soc. Neurosci..

[76] Ly P.T.T., Wu Y., Zou H., Wang R., Zhou W., Kinoshita A., Zhang M., Yang Y., Cai F., Woodgett J. (2013). Inhibition of GSK3β-Mediated BACE1 Expression Reduces Alzheimer-Associated Phenotypes. J. Clin. Investig..

[77] Kuhla A., Ludwig S.C., Kuhla B., Münch G., Vollmar B. (2015). Advanced Glycation End Products Are Mitogenic Signals and Trigger Cell Cycle Reentry of Neurons in Alzheimer’s Disease Brain. Neurobiol. Aging.

[78] Langlais P.J., Anderson G., Guo S.X., Bondy S.C. (1997). Increased Cerebral Free Radical Production during Thiamine Deficiency. Metab. Brain Dis..

[79] Tebay L.E., Robertson H., Durant S.T., Vitale S.R., Penning T.M., Dinkova-Kostova A.T., Hayes J.D. (2015). Mechanisms of Activation of the Transcription Factor Nrf2 by Redox Stressors, Nutrient Cues, and Energy Status and the Pathways through Which It Attenuates Degenerative Disease. Free Radic. Biol. Med..

[80] Bozic I., Savic D., Stevanovic I., Pekovic S., Nedeljkovic N., Lavrnja I. (2015). Benfotiamine Upregulates Antioxidative System in Activated BV-2 Microglia Cells. Front. Cell. Neurosci..

[81] Hansen D.V., Hanson J.E., Sheng M. (2018). Microglia in Alzheimer’s Disease. J. Cell Biol..

[82] Hazell A.S., Butterworth R.F. (2009). Update of Cell Damage Mechanisms in Thiamine Deficiency: Focus on Oxidative Stress, Excitotoxicity and Inflammation. Alcohol. Alcohol..

[83] Todd K.G., Butterworth R.F. (1999). Early Microglial Response in Experimental Thiamine Deficiency: An Immunohistochemical Analysis. Glia.

[84] Sanchez-Ramirez G.M., Caram-Salas N.L., Rocha-Gonzalez H.I., Vidal-Cantu G.C., Medina-Santillan R., Reyes-Garcia G., Granados-Soto V. (2006). Benfotiamine Relieves Inflammatory and Neuropathic Pain in Rats. Eur. J. Pharmacol..

[85] Yadav U.C.S., Kalariya N.M., Srivastava S.K., Ramana K.V. (2010). Protective Role of Benfotiamine, a Fat-Soluble Vitamin B1 Analogue, in Lipopolysaccharide-Induced Cytotoxic Signals in Murine Macrophages. Free Radic. Biol. Med..

[86] Bozic I., Savic D., Laketa D., Bjelobaba I., Milenkovic I., Pekovic S., Nedeljkovic N., Lavrnja I. (2015). Benfotiamine Attenuates Inflammatory Response in LPS Stimulated BV-2 Microglia. PLoS ONE.

[87] Shoeb M., Ramana K.V. (2012). Anti-Inflammatory Effects of Benfotiamine Are Mediated through the Regulation of the Arachidonic Acid Pathway in Macrophages. Free Radic. Biol. Med..

[88] Vatsalya V., Li F., Frimodig J., Gala K.S., Srivastava S., Kong M., Ramchandani V.A., Feng W., Zhang X., McClain C.J. (2021). Repurposing Treatment of Wernicke-Korsakoff Syndrome for Th-17 Cell Immune Storm Syndrome and Neurological Symptoms in COVID-19: Thiamine Efficacy and Safety, In-Vitro Evidence and Pharmacokinetic Profile. Front. Pharmacol..

[89] Clayton D.A., Grosshans D.R., Browning M.D. (2002). Aging and Surface Expression of Hippocampal NMDA Receptors. J. Biol. Chem..

[90] Heywood R., Wood J.D., Majeed S.K. (1985). Tumorigenic and Toxic Effect of O,S-Dibenzoyl Thiamine Hydrochloride in Prolonged Dietary Administration to Rats. Toxicol. Lett..

[91] Ketola H.G., Isaacs G.R., Robins J.S., Lloyd R.C. (2008). Effectiveness and Retention of Thiamine and Its Analogs Administered to Steelhead and Landlocked Atlantic Salmon. J. Aquat. Anim. Health.

[92] Safavi M., Hosseini-Sharifabad A., Seyed-Yousefi Y., Rabbani M. (2020). Protective Effects of Citicoline and Benfotiamine Each Alone and in Combination on Streptozotocin-Induced Memory Impairment in Mice. Clin. Psychopharmacol. Neurosci. Off. Sci. J. Korean Coll. Neuropsychopharmacol..

[93] Putnam E.E., Goodman A.L. (2020). B Vitamin Acquisition by Gut Commensal Bacteria. PLoS Pathog..

[94] Costliow Z.A., Degnan P.H. (2017). Thiamine Acquisition Strategies Impact Metabolism and Competition in the Gut Microbe Bacteroides Thetaiotaomicron. mSystems.

[95] Fung T.C., Olson C.A., Hsiao E.Y. (2017). Interactions between the Microbiota, Immune and Nervous Systems in Health and Disease. Nat. Neurosci..

